# Phlorotannins and glycolipid metabolism: comprehensive regulatory roles mediated by the gut microbiota

**DOI:** 10.3389/fnut.2026.1750434

**Published:** 2026-02-03

**Authors:** Shuyan Wu, Ziqi Sui, Jie Pan, Xiao Men, Xiang Li, Dongping Xue, Qingbao Meng, Xionggao Han, Yimin Shen

**Affiliations:** 1 The Second Clinical Medical College, Zhejiang Chinese Medical University, Hangzhou, Zhejiang Province, China; 2 Department of Gastroenterology, The Second Affiliated Hospital, Zhejiang Chinese Medical University, Hangzhou, Zhejiang Province, China; 3 Department of Endocrinology, The Second Affiliated Hospital of Zhejiang University, Hangzhou, Zhejiang Province, China; 4 Qingdao Mingyue Seaweed Group Co.Ltd. National Key Laboratory of Marine Food Processing and Safety Control, Qingdao, Shandong Province, China; 5 Jinhua Institute of Zhejiang University, Zhejiang University, Jinhua, China; 6 School of Pharmacy, Zhejiang University, Hangzhou, Zhejiang Province, China

**Keywords:** gut microbiota, metabolism, phloroglucinol, phlorotannins, polyphenols

## Abstract

Metabolic disorders are precursors to numerous chronic diseases. Phlorotannins is a kind of natural bioactive agents found in brown algae composed with polyphenolic. Due to its role in regulating blood glucose and lipid levels, it is expected to manage chronic metabolic disease. However, previous study has mainly focused phlorotannins on their metabolic effects in the native state. Due to their high molecular weight, these compounds are poorly absorbed in the intestine, which limits their oral bioavailability. This review examines the interactions between phlorotannins and gut microbiota, as well as the role of small-molecule metabolites produced by microbial degradation on host metabolism. Phlorotannins can modulate the composition of the gut microbiota, promote the production of short-chain fatty acids, and increase bile acid metabolism. Therefore, understanding the bioavailability of gut microbiota-derived phlorotannin metabolites is crucial for developing strategies to prevent obesity and manage diabetes.

## 1 Introduction

Obesity, which is defined as a body mass index (BMI) > 30 kg/m^2^, is among the primary risk factors for metabolic disorders. The global obesity rate is projected to surpass 6% in men and 9% in women by 2025 (1). Obesity contributes to increased risks of type 2 diabetes mellitus (T2DM), cardiovascular disease (CVD), hypertension, nonalcoholic fatty liver disease (NAFLD), neurodegenerative diseases (2), and certain cancers. Among these, T2DM is the most common type of diabetes, accounting for approximately 90% of all cases (3). By 2035, population with type 2 diabetes is expected to reach 592 million (4), which puts a growing health burden on the world. The maintenance of stable postprandial blood glucose is vital for health. Chronic postprandial hyperglycemia drives increased glycated hemoglobin levels, contributing to the development of diabetes, lipid metabolism disorders, and CVD (5). Current pharmacological treatments for these conditions can control disease progression but are often associated with adverse effects. The management of metabolic diseases typically involves dietary interventions, physical exercise, and the hypoglycemic and lipid-lowering drugs. However, these medications frequently cause gastrointestinal side effects. Consequently, exploring natural alternatives to improve the quality of life for affected individuals has attracted growing interest.

Over the past few decades, marine algae resources have attracted attention for their bioavailable value because of their potential in the food, cosmetics, and pharmaceutical industries. Various secondary metabolites of algae have been investigated for their diverse functional properties, such as antioxidant (6), anti-inflammatory (7, 8), anticancer (9), antidiabetic (10), antibacterial (11), immunomodulatory, and antihypertensive effects (12). These metabolites include polyphenols, polysaccharides, terpenoids, alkaloids, polyunsaturated fatty acids, proteins, peptides, amino acids, and halogenated derivatives of polyphenols. Tannins are a unique class of phenolic metabolites with molecular weights ranging from 500 to 30,000 Da and are widely distributed in almost all plant-based foods and beverages (13).

Recent studies have shown that polyphenols from marine organisms have remarkable pharmacological potential in metabolic disorders (14). Algal polyphenols include phenolic acids, phlorotannins, flavonoids, and halogenated derivatives (15). Phlorotannins, a class of polyphenols derived from brown algae, have gained significant interest due to their strong antibacterial and cytotoxic activities. In addition, their wide distribution in temperate and polar marine environments make them a promising source of marine biomass. However, the high molecular weight of these compounds limits their direct absorption and oral bioavailability. Nevertheless, recent studies show that the gut microbiota can degrade phlorotannins, which increases their pharmacological activity and helps improve metabolic health in the host (16). The gut microbiota, a complex community of microorganisms residing in the colon, plays a fundamental role in human physiology (17). For example, the gut microbiota regulates host energy homeostasis, including energy production, storage, and expenditure (18). Since intestinal glucose absorption is influenced by the gut environment and glucose transport rates, it is critical to understand how phlorotannins affect the processes, including digestive enzyme activity and glucose transport, to better elucidate their mechanism in regulating postprandial blood glucose. Studies have shown that extra seaweed into the diet can change the diversity of the gut microbiota in the host, which suggest bioactive phenolic compounds may be related to the microbial changes.

This review aims to: (i) summarize the species and distribution of brown algae while comparing the properties of marine-derived phlorotannins with terrestrial polyphenols; (ii) analyze the role of the gut microbiota in enhancing the bioavailability of phlorotannins; (iii) systematically elucidate the small-molecule metabolites derived from microbial degradation of phlorotannins and their functions in metabolic regulation; and (iv) summarize the current evidence from preclinical and clinical studies on the metabolic effects of phlorotannins.

## 2 Sources, structural diversity and unique traits of phlorotannins

### 2.1 Species and distribution of brown algae (Phaeophyceae)

Marine ecosystems are rich sources of bioactive compounds. Seaweeds are major producers in marine environments and are sources of various bioactive compounds, accounting for 40% of global photosynthesis (19). There are approximately 10,000 species of seaweed worldwide, classified as brown, red or green algae on the basis of their pigmentation. Brown seaweeds contain higher concentrations of bioactive components than red and green seaweeds do (20). Among brown seaweed species, *Ascophyllum nodosum* and *Fucus vesiculosus* exhibit the highest antioxidant values and total phenolic content (21).

The Phaeophyceae family (*Fucaceae*) is a dominant algal group in the intertidal zones of cold to warm-temperate Northern Hemisphere regions, encompassing genera such as *Ascophyllum, Fucus, Pelvetia, Pelvetiopsis,* and *Silvetia.* Among these, *Fucus* is the most prominent and widely distributed genus, comprising 66 recognized species. The dominant phlorotannins in *Fucus* spp. are fucophlorethols, characterized by molecular weights of 370–746 Da and a relatively low degree of polymerization (3–6 phloroglucinol units, PGU) (22). Studies have reported that the ethyl acetate extract of *Ecklonia cava* (EC-ETAC) exerts anti-obesity effects on 3 T3-L1 preadipocytes via the HO-1/Nrf2 pathway (23). Specifically, EC-ETAC significantly suppressed the expression of key adipogenic transcription factors (PPARγ, C/EBPα, and SREBP-1) and related proteins (FAS and LPL), indicating its role in promoting lipolysis and brown adipose tissue formation. Furthermore, within the Phaeophyceae family, *Ascophyllum* and *Pelvetia* are monotypic genera endemic to the North Atlantic, represented by *A. nodosum* (knotted wrack) and *P. canaliculata* (channeled wrack), respectively. In contrast, *Pelvetiopsis* and *Silvetia* are endemic to the North Pacific (24–26).

**Figure 1:**
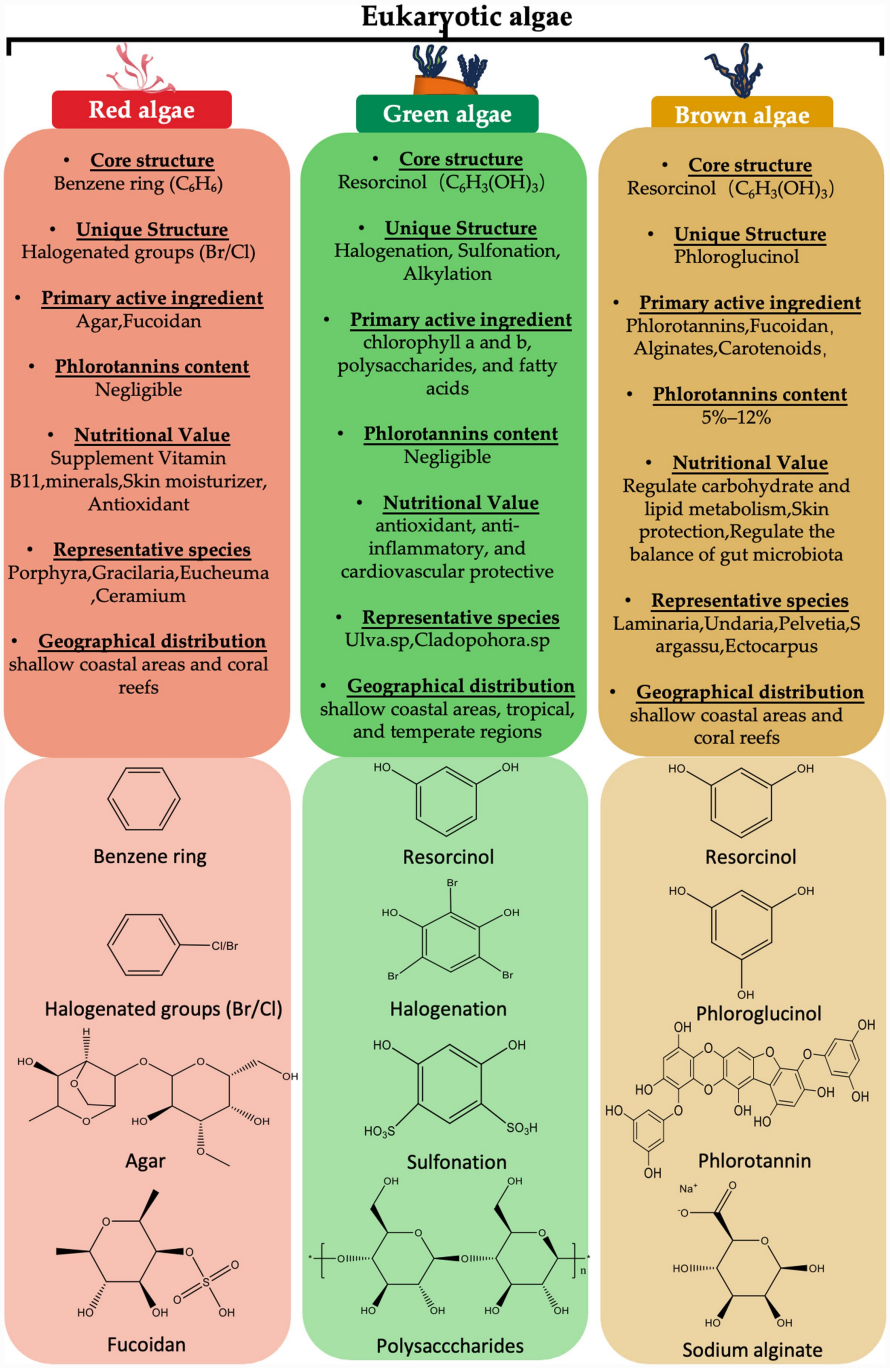
From structure to function: an overview of major algal groups and species.

Algae produce a wide spectrum of secondary metabolites, including polyphenols, polysaccharides, terpenoids, alkaloids, and halogenated derivatives (26). Commonly identified polyphenols encompass phlorotannins, catechins, bromophenols, and fucoxanthin, among others (27). In particular, brown algae are distinguished by their high phlorotannin content, which can reach 25% of dry weight, whereas red and green algae predominantly contain phenolic acids and flavonoids (28, 29). Remarkably, although most brown algae synthesize phlorotannins with up to 39 phloroglucinol units, *P. canaliculata* produces polymers of up to 49 units—possibly related to its adaptation to extreme habitats (30). As shown in Figure 1, the major algal groups display characteristic structural and functional features that define their biological roles across species.

### 2.2 Structural diversity of phlorotannins

As the main polyphenolic compounds in brown algae (orders *Laminariales* and *Fucales*) (31), phlorotannins are hydrophilic polymers of phloroglucinol with molecular weights from 126 Da to 650 kDa (32). Their biosynthesis follows the acetate–malonate pathway, initiated by type III polyketide synthase converting acetyl-CoA to malonyl-CoA and culminating in phloroglucinol formation through cyclization and tautomerization. Variations in polymerization degree, linkage patterns, and substituents generate substantial structural diversity, with approximately 150 isomers reported to date (15). For precise structural characterization, nuclear magnetic resonance (NMR) combined with high-resolution mass spectrometry is the optimal approach (33). Low-molecular-weight phlorotannins are classified in Table 1.

**Table 1:** Structure, distribution and metabolic characteristics of low-molecular-weight brown algal polyphenols.

Category	Fucols	Phlorethols	Fucophlorethols
Core chemical skeleton	Resorcinol units polymerize via C-C bonds (ortho/para)	The resorcinol unit polymerizes via C-O-C ether bonds	Resorcinol units are connected via a mixture of C-C and C-O-C ether bonds
Key structural features	Contains only C-C bonds, no ether bonds; Phenolic hydroxyl groups are distributed at the ortho and para positions of the benzene ring, resulting in high rigidity	Contains only ether bonds, exhibiting high structural symmetry; Molecular chain flexibility surpasses that of Fucols	Contains mixed bonds, highly branched; Uneven distribution of phenolic hydroxyl groups
Typical DP	2–10 (up to 15 in some cases)	3–12 (partial coverage up to 20)	5–30 (up to 50 in some cases)
Representative sources	*Fucusvesiculosus, Ascophyllumnodosum*	*Ecklonia cava, Fucus serratus*	*Fucus distichus, Laminaria hyperborea*
Relationship with gut microbiota	Gut microbiota esterases exhibit low sensitivity to its degradation, requiring slow breakdown by C-C bond cleavers secreted by Bacteroides species; It promotes proliferation of SCFA-producing bacteria; Inhibiting pathogenic	Rapidly degraded by ether bond-cleaving enzymes secreted by gut microbiota, yielding metabolites such as gallic acid and p-hydroxybenzoic acid; Modulates microbial composition: increases Bifidobacterium abundance while reducing Desulfovibrio proportion	Highly branched structure binds to surface polysaccharides of gut microbiota, enhancing intestinal adhesion of bacteria like *Bacteroides fragilis*; Requires synergistic degradation by multiple microbial species, with degradation products primarily comprising SCFAs and phenolic acids
Category	Fuhalols	Eckols	Halogenated phlorotannins
Core chemical skeleton	The resorcinol unit is connected via a para-C-C bond and contains a sulfonate group (-SO₃H)	Oligomerized structure based on eckol (dimer, diether-linked six-membered ring) as the monomer unit (101)	Using any class (e.g., phlorethols, eckols) as the parent compound, the benzene ring contains Cl/Br substitution
Key structural features	C-C bond in a linear structure; sulfate ester group enhances polarity	Contains the characteristic “eckol ring,” primarily oligomers (DP = 2–3); phenolic hydroxyl groups are fully exposed	Halogen substitution (1–2 per molecule), parent skeleton determines basic structure; enhanced hydrophobicity
Typical DP	2–6 (rare high DP)	2–3	2–10 (same as parent)
Representative sources	*Fucus vesiculosus, Pelvetia squamosa*	*Ecklonia cava*, *Ecklonia stolonifer*	*Asparagopsis armata*, *Sargassum fusiform*
Relationship with gut microbiota	Sulfate groups can bind to surface proteins of gut microbiota, modulating the activity of microbial metabolic enzymes; The degradation process releases sulfate ions, inhibiting the activity of bile salt hydrolase (BSH) within the intestine	Small molecular weight (low DP), partially absorbed directly by the small intestine; the unabsorbed portion is degraded by *Bacteroides ovatus* into phloroglucinol; Directly inhibits the quorum sensing system of intestinal pathogens, reducing toxin secretion	Halogen atoms disrupt the cell membrane integrity of intestinal pathogens, selectively inhibiting their growth while minimally affecting beneficial bacteria; Degrades the dehalogenase enzymes relied upon by bacteria like *Clostridium perfringens*, generating non-halogenated active compounds

Brown algal species exhibit substantial variation in phlorotannin content. Extracts from *Fucales* species range between 145.11 and 275.97 μg PGE/100 mg dry extract, following the order *F. serratus* > *F. guiryi* > *F. spiralis* > *F. vesiculosus* (34), whereas *Sargassum tenerrimum* shows a notably high level of 10.00 mg phloroglucinol/g (30). This variability is shaped by multiple factors such as algal morphology, developmental stage, tissue type, and abiotic conditions, such as salinity, light and temperature. Furthermore, a pronounced latitudinal gradient exists: high-latitude populations often contain >4% DW phlorotannins, compared to <2% DW in low-latitude counterparts, suggesting an adaptive response to environmental stimuli (35).

Phlorotannins play essential roles in brown algae growth and survival. They participate in maintaining their cell wall integrity, protecting ultraviolet hurt, and resisting herbivores invasion. Certain phlorotannins are halogenated (bromine/chlorine/iodine) and exhibit a bitter taste while inhibiting digestive enzymes such as amylase and trypsin in grazers. For instance, *Fucus* increases phlorotannin synthesis in summer, reducing blade grazing by over 40%. In particular, phlorotannins protect against UV-induced damage: under UV stress, soluble phlorotannin levels vary with antioxidant activity, while insoluble pools remain constant (36). This indicates that phlorotannins scavenge free radicals, quench UV-induced ROS, protect photosynthetic enzymes and mitigate oxidative stress, which help brown algae thrive in the intertidal zone environment.

### 2.3 Unique characteristics of phlorotannins compared with those of terrestrial polyphenols

More than 8,000 structurally distinct polyphenols have been identified from terrestrial plants and marine algae (37). Terrestrial polyphenols mainly come from seeds, roots, bark and stems, and are rich in cocoa, tea, fruits and beans. Their extraction is well-established, and studies have demonstrated their efficacy against metabolic disorders. For instance, rutin can ameliorate gut microbiota dysbiosis in diabetic mice by modulating specific bacterial genera (38, 39). In comparison, marine polyphenols, particularly phlorotannins from brown algae, offer distinct advantages. Their content in seaweeds can be 10–100 times higher than in terrestrial foods (40). The unique interphenyltriol structure (40) exhibites with strong anti-inflammatory activity by inhibiting pro-inflammatory cytokines (41), and can reduce liver fat degeneration by enhancing fatty acid β-oxidation (42). Marine polyphenols are distinguished by their electron-rich structures, which are often functionalized with hydroxyl groups, ether bonds and halogen substituents. This structural signature enhances their ability to scavenge free radicals and bind biological targets (43, 44). Together with their capacity to promote probiotic growth, these attributes highlight considerable promise for use in therapeutics and cosmeceuticals. Nevertheless, practical exploitation remains challenging due to difficulties in reliable sourcing and efficient extraction. As summarized in Table 2, polyphenols from terrestrial and marine sources differ markedly in their structural features, bioactivities, and physiological functions.

**Table 2:** Comparison of polyphenols from terrestrial and marine sources.

Comparative perspective	Terrestrial polyphenols	Brown algae polyphenols
Core chemical structure	Featuring a C6-C3-C6 (flavonoid) or C6-C1/C2 (phenolic acid/quinone) backbone; Exhibiting significant variation in polymerization degree, with a high proportion of low-polymerization monomers	Using phloroglucinol as the basic unit; Typically exhibit high polymerization degrees (DP = 2–50), with certain classes
Gut microbiota dependency	Partial dependence: Low-polymerization monomers can be directly absorbed by the small intestinewithout requiring microbial degradation	Complete dependence: High polymerization degree + specific functional groups result in a small intestine direct absorption rate <5%, requiring nearly complete degradation by colonic microbiota (50)
Gut microbiota metabolites	Primarily composed of a single type of small-molecule phenolic acid	Generate a mixture of phenolic acids, specific active molecules, high-abundance SCFAs
Regulation of metabolism in the body	Glucose metabolism: Mild stimulation of GLP-1 secretion via SCFAs or direct monomer activation of the AMPK pathway reduces fasting blood glucose levels; Lipid Metabolism: Inhibits fatty acid synthase (FAS) to reduce triglyceride production, or lowers serum LDL-C by 5–10% through weak estrogenic activity	Glucose Metabolism: Phloroglucinol inhibits hepatic gluconeogenic enzymes (PEPCK), increases GLP-1 secretion (45); Lipid Metabolism: Sulfate esters inhibit bile acid synthase (BSH), promoting bile acid excretion. Serum LDL-C decreases by 15–20%, with lipid-lowering effects 2–3 times greater than terrestrial polyphenols

## 3 Gut microbiota-mediated biotransformation of phlorotannins

### 3.1 Absorption characteristics of phlorotannins

Bioavailability of phenols refers to the proportion of ingested compounds that reach the systemic circulation to exert biological effects, serving as a key indicator of bioefficacy. Phlorotannins generally exhibit low bioavailability, ranging from 2 to 14% (45). While small-molecule forms are more readily absorbed, their high-molecular-weight counterparts primarily function as a physical UV barrier. However, these macromolecular polymers (often >100 kDa) can be degraded into active fragments. After ingestion, only about 14.1% of *Fucus vesiculosus*-derived phlorotannins are absorbed in the upper gastrointestinal tract (46), consistent with earlier findings (6). The majority accumulate in the colon, where gut microbiota can metabolize them into absorbable small molecules. This is supported by urinary and plasma metabolite profiling: most phlorotannin metabolites are detected 6–24 h post-consumption, confirming limited small-intestinal absorption and predominant colonic processing (47, 48).

**Figure 2:**
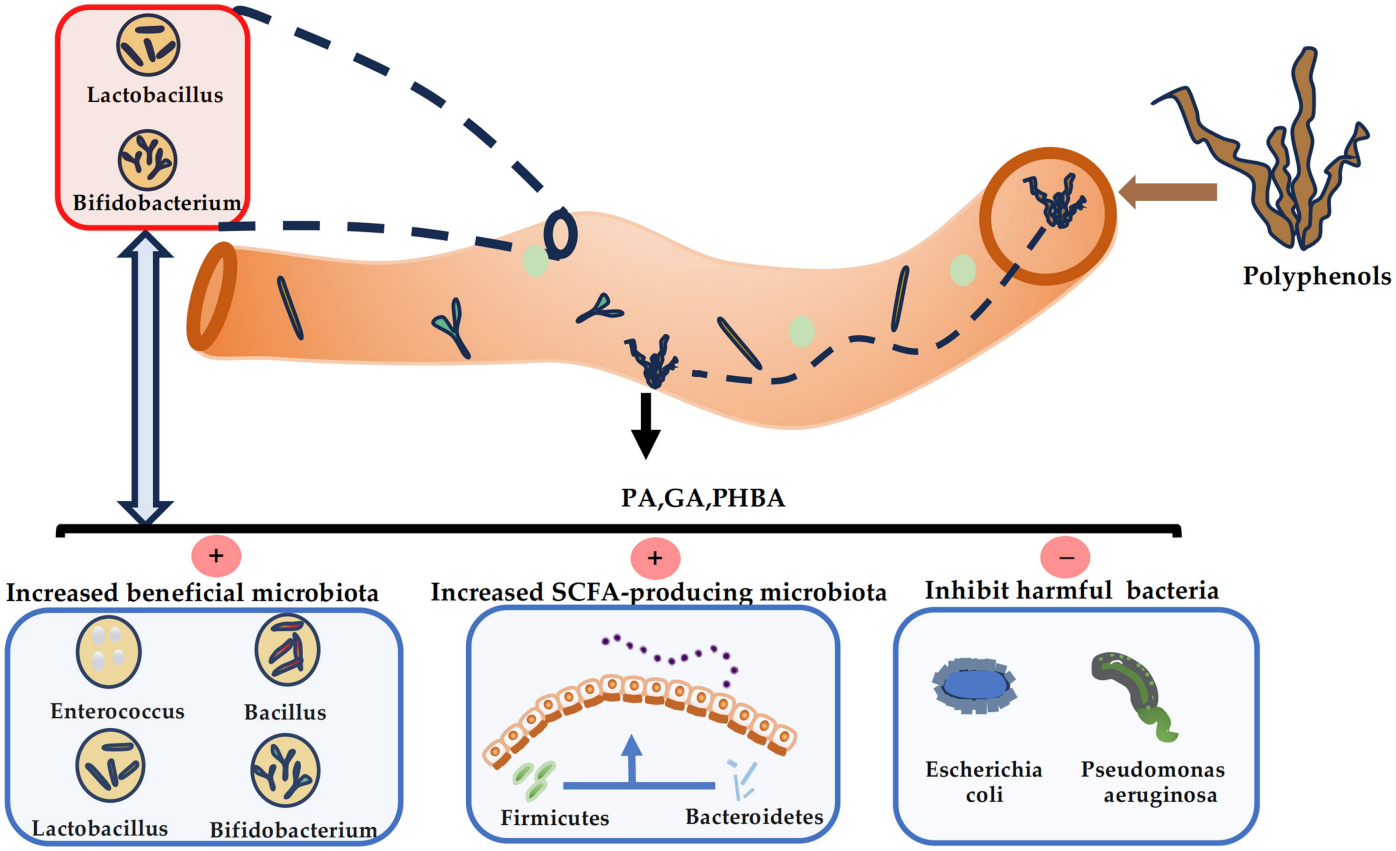
Interplay between brown algae polyphenols and gut microbiota. Phlorotannins are effectively catabolized by lactic acid bacteria and bifidobacteria into low-molecular-weight metabolites. These microbial derivatives subsequently restructure the gut community by enriching beneficial, short-chain fatty acid producing bacteria and suppressing potential pathogens.

The bioavailability of phlorotannins, which is crucial for their efficacy, is predominantly governed by gut microbiota. A small fraction of low-molecular-weight phlorotannins may be directly absorbed by small intestinal epithelial cells and undergo conjugation in the intestinal mucosa or liver, forming glucuronidated, sulfated, or methylated derivatives that influence their polarity and initial bioavailability (43). However, the majority of these polymers resist upper gastrointestinal absorption and instead transit to the colon, where they are degraded by microbial enzymes, like polyphenol oxidases and hydrolases into smaller phenolic acids (49). These metabolites are modified by host enzymes, including cytochrome P450, glucuronosyltransferases and sulfotransferases in the liver and kidneys before excretion (50). This microbial-centric metabolic pathway is supported by following evidence. For example, seaweed supplementation has been shown to enrich beneficial bacteria such as *Shewanella* sp. in fish models, improving intestinal barrier function (48). Similarly, the rapid appearance of gallic acid in humans within 2 h post-ingestion suggests efficient hydrolysis by tannin-degrading genera like *Lactobacillus* and *Bifidobacterium* (51). Later-stage colonic metabolites, such as 2,3-dihydroxybenzoic acid, may involve hydroxylation by *Bacillus* or *Enterococcus* species (25). These findings align with the established role of phenolic compounds in modulating gut microbiota. However, future studies are needed to fully elucidate the specific microbial taxa and enzymatic pathways involved in phlorotannin metabolism. The interaction between brown algal polyphenols and the gut microbiota is summarized in Figure 2.

### 3.2 Biotransformation pathways of phlorotannins

The human gut microbiota exhibits distinct compositional and functional gradients along the gastrointestinal tract. Due to the acidic environment in stomach, the microbial community is relatively sparse; whereas, the large intestine is the main place for microbial colonization and metabolic activities (52). This is also the primary site for the extensive degradation of phlorotannins, a process orchestrated by bacterial enzymes from the gut microbiota. Through a stepwise enzymatic process involving polyphenol esterases and ether bond-cleaving enzymes from genera such as Bacteroides and Lactobacillus, macromolecular phlorotannins are first depolymerized into oligomers like eckol. These are further cleaved into monomers like phloroglucinol by Clostridium and other genera, and finally converted into bioavailable phenolic acids, such as gallic acid and p-hydroxybenzoic acid via dehydroxylases (53).

**Figure 3:**
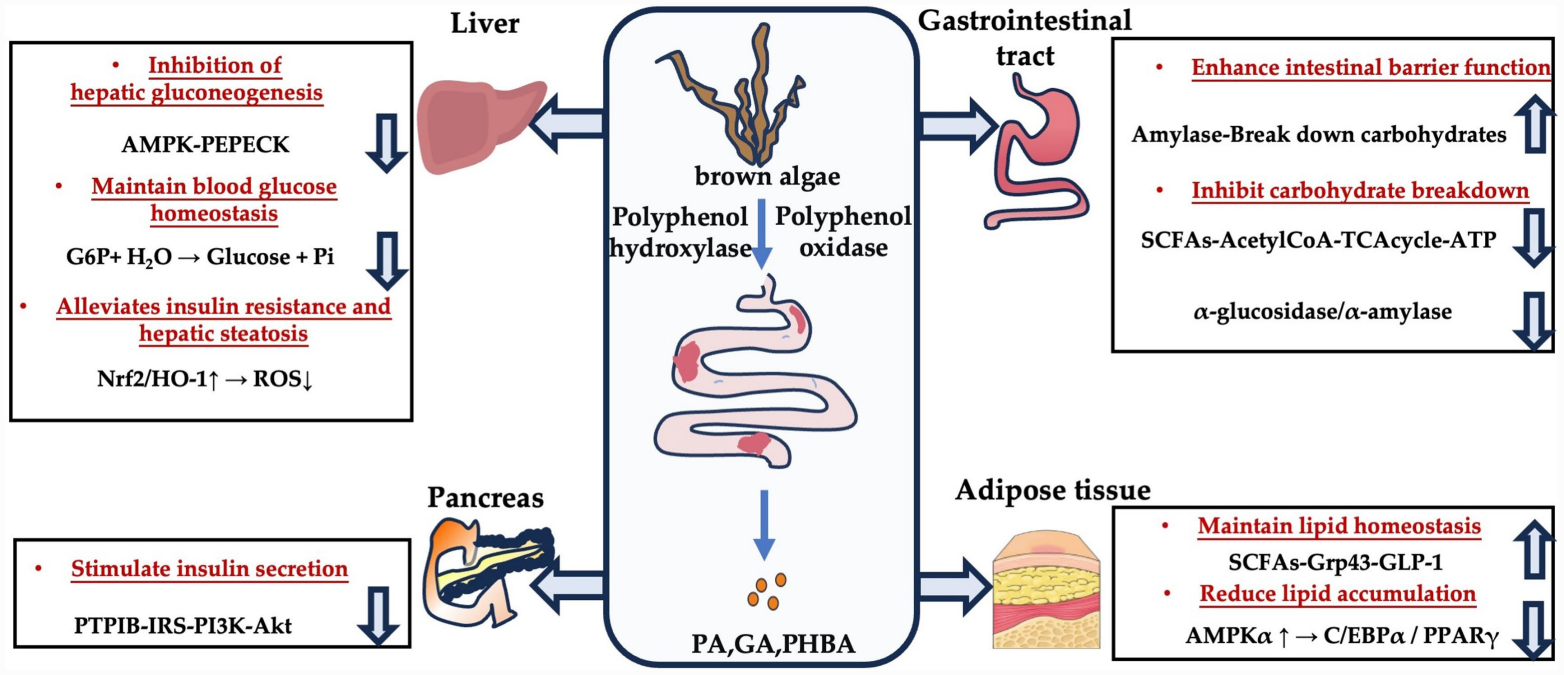
Mechanisms of phlorotannin metabolites in regulating human metabolism. Schematic overview of the role of phlorotannins and their microbial metabolites (PA, GA, PHBA) in glucose and lipid metabolism. Gut microbiota-derived polyphenol oxidases and hydrolases convert phlorotannins into bioactive small molecules. At the hepatic level, these metabolites inhibit gluconeogenesis through AMPK/PEPCK signaling and G6P suppression, while enhancing insulin sensitivity via IRS/PI3K/AKT activation and PTP1B inhibition. In parallel, activation of the Nrf2/HO-1 antioxidant axis reduces reactive oxygen species (ROS) accumulation, thereby alleviating oxidative stress–driven insulin resistance and hepatic lipid peroxidation. In the gut, they attenuate carbohydrate digestion by inhibiting α-glucosidase and α-amylase, and promote a favorable microbiome that produces SCFAs. SCFAs activate GPR43, stimulating lipolysis and energy expenditure. In the pancreas, these metabolites stimulate insulin secretion via the PTP1B/IRS/PI3K/Akt signaling pathway. In adipose tissue, they maintain lipid homeostasis through the SCFAs/GPR43/GLP-1 signaling axis, contributing to glucose and lipid metabolic regulation. Additionally, phlorotannin-associated activation of AMPKα suppresses adipogenic transcription factors C/EBPα and PPARγ, thereby inhibiting adipogenesis and limiting lipid accumulation.

Together with phlorotannin-stimulated SCFAs, these microbial metabolites form key regulators in host metabolism. This degradative capability represents an evolutionary adaptation to dietary polyphenols. Although phlorotannins possess antibacterial properties due to their phenolic hydroxyl groups, which can disrupt bacterial membranes (54), certain microbiota encodes detoxifying enzymes, including β-glucosidase and O-demethylase, these enzymes not only neutralize the toxicity of phlorotannins but also use them as carbon source, giving these microorganisms a competitive advantage. Consequently, the degradation process rebuilds the gut microbial structure: enriching beneficial degraders and SCFA-producers (*Faecalibacterium prausnitzii* and Roseburia) while suppressing pathogens. Therefore, elucidating the key degradative pathways will be crucial to clarify the “gut microbiota-metabolites-host” axis and uncover the underlying mechanisms of phlorotannins in modulating health. The metabolic pathways through which phlorotannin-derived metabolites regulate glucose and lipid homeostasis are summarized in Figure 3.

### 3.3 Comparison of bioactivity between parent phlorotannins and gut-derived metabolites

Research on phlorotannins relies on accurate quantitative analytical methods. Current techniques include spectrophotometric assays, such as the Folin–Ciocalteu method, high-performance liquid chromatography (HPLC), and nuclear magnetic resonance (NMR), which provide a reliable basis for the study of metabolic processes of phlorotannins. At the metabolic level, the enzymatic degradation of phlorotannins by the gut microbiota represents a central link in their bioactivity. Specific microbial enzymes decompose macromolecular polyphenols into various of small molecule metabolites These metabolites have pharmacological properties differed from their parent compounds, and are the main active forms of PTs that exerts health benefits. Indeed, the physiological functions of polyphenols are largely determined by these microbially derived bioactive metabolites (55).

The underlied mechanism of this phenomenon is that low-molecular-weight metabolites usually have higher intestinal permeability and bioapailability, so that they can effectively enter the body circulation, reach the target, and give full play to their physiological activity. In contrast, high-molecular-weight polyphenols are difficult to pass through the intestinal barrier and show minimal biovailability (56). It should be noted that interindividual variations in gut microbiota composition directly influence the types and proportions of polyphenol metabolites produced (57). For example, individuals with healthy intestinal flora (rich in beneficial bacteria such as Mycobacterium and Bifidobacterium) are more likely to convert polyphenols into anti-inflammatory compounds, such as procatechic acid (58). On the contrary, in the case of intestinal flora disorder, the metabolic spectrum may turn to prosuce inactive by-products like Phenylpropionic acid. Therefore, to maintain a healthy intestinal microbial ecosystem is crucial to make polyphenol produce highly active, low molecular weight phenolic metabolites, thus, to improve their health benefits. Intervention studies in humans or rats are commonly used to track the metabolic pathways of polyphenols. The analysis of blood, urine and fecal samples after ingestion helps clarify microbiota-derived metabolites and map their pathways and distribution.

Moreover, results from *in vitro* experiments-such as co-culture of gut microbiota with polyphenols -confirm that the microbial catabolism of phlorotannins not only alters their original bioactivity but also generates highly bioavailable active metabolites (59). Specifically, phlorotannins are primarily degraded into low-molecular-weight metabolites such as phenolic acids and SCFAs. The metabolites exhibit higher bioavailability than their native precursors and exert more potent regulatory effects on host metabolism. For instance, gallic acid helps modulate blood glucose by inhibiting hepatic gluconeogenesis (60). Meanwhile, SCFAs activate intestinal receptors GPR43 and GPR41, which promote lipid breakdown and help maintain body weight and lipid balance (61). Notably, besides phlorotannins, other brown algae-derived compounds such as alginate and fucoxanthin also exhibit substantial physiological activity (62). Studies indicate that all three components effectively inhibit lipase activity, suggesting a complementary role in reducing dietary fat digestion and absorption.

### 3.4 Potential synergistic effects between phlorotannins and other bioactive compounds

In brown seaweeds, phlorotannins inherently co-occur with algal polysaccharides (6), particularly alginates and fucoidans, as well as dietary fibers (63) and minor bioactive compounds (64). Alginates are linear polysaccharides primarily composed of mannuronic acid and guluronic acid units (65). Alginates play a key role in the structural integrity of the cell walls. Fucoidan is a sulfated polysaccharide mainly composed of fucose along with other sugars like galactose, xylose, and mannose. It is known for its immune-modulating, anti-coagulant, and anti-inflammatory properties (66). Phlorotannins and alginates are commonly present together in the cell walls of brown algae, forming a complex network of bioactive compounds that interact with each other. These interactions are primarily due to the chemical nature of these components, where phlorotannins, as strong antioxidants, can form high molecular weight complexes with alginates under the action of oxidative enzymes (67).

Several biochemical and structural studies have indicated that (68), phlorotannins can undergo oxidative cross-linking with polysaccharides such as alginates to form high-molecular-weight complexes, thereby modulating the sequestration and release of bioactive compounds. This physicochemical association represents an intrinsic form of synergistic interaction between phlorotannins and algal polysaccharides. Similarly, phlorotannins and alginate likely suppressed microbial activity, thereby slowing the increase in pH. Consistent with this notion, a recent study demonstrated that a phlorotannin–alginate combination from brown algae synergistically inhibited polyphenol oxidase activity, with 2% phlorotannins + 1% alginate achieving the highest inhibition (84.51%), comparable to 1% ascorbic acid (72.43%), and effectively delayed melanosis and overall quality deterioration in ice-stored Pacific white shrimp (69).

These complexes enhance the stability and bioavailability of the compounds, potentially affecting nutrient absorption, metabolism, and immune modulation. For instance, alginates and fucoidans are known to modulate gut microbiota and promote short-chain fatty acid production (70), while phlorotannins contribute by further enhancing these effects through antioxidant and anti-inflammatory mechanisms. The combined action of these compounds may regulate various metabolic processes, such as lipid and glucose metabolism, by improving gut barrier function, modulating gut microbiota composition, and enhancing nutrient absorption (71).

## 4 Mechanisms underlying the regulation of host metabolism by phlorotannins via the gut microbiota

Phlorotannins directly modulate metabolic processes by targeting key enzymes and pathways involved in glucose and lipid homeostasis. In carbohydrate metabolism, they inhibit *α*-glucosidase and α-amylase (72), which slows down carbohydrate digestion and postprandial glucose absorption. They also suppress protein tyrosine phosphatase 1B (PTP1B) activity, thereby enhancing insulin signaling and sensitivity (73). They also downregulate the expression of liver enzymes involved in gluconeogenesis, such as glucose-6-phosphatase (G6Pase) and phosphoenolpyruvate carboxykinase (PEPCK), while increasing the activity of glucokinase (GK), thus promoting glucose utilization and storage. Based on mouse and zebrafish models in lipid metabolism, *Dieckol* could activate AMPKα signaling to suppress lipid accumulation (74). Other compounds, such as those derived from *Eucalyptus cavaleriei*, *Crepidotus applanatus*, and *Ishige okamurae*, similarly inhibit adipogenesis by downregulating C/EBPα and PPARγ and inducing preadipocyte apoptosis (75). Taken together, these findings underscore the established role of phlorotannins in regulating metabolism.

Since gut microbiota was first identified in host energy homeostasis in 2004 (76), its contribution to metabolic regulation has been widely recognized. Phlorotannins are poorly absorbed in the upper digestive tract, and rely heavily on microbial transformation to produce bioavailable metabolites such as GA and PHBA. Beneficial bacteria including Bifidobacterium and Bacteroides are primarily responsible for this conversion. And phlorotannins modulate gut microbial composition in return; for instance, *Sargassum* polyphenol extracts inhibit biofilm formation in opportunistic pathogens like *Escherichia coli* and *Pseudomonas aeruginosa* (77), thereby promoting microbial balance and facilitating efficient phlorotannin transformation.

The following sections will focus on how the small molecule metabolites of phlorotannins affect host metabolism and interact with gut microbial community.

### 4.1 Regulation of glucose metabolism

*In vitro* studies have demonstrated that a specific phlorotannin extract dose dependently inhibits α-amylase and α-glucosidase. Consistent with these results, animal studies have shown that the same extract can reduce postprandial blood glucose peaks by 90% and insulin secretion peaks by 40%. These results collectively confirm the efficacy of this extract in modulating carbohydrate digestion and absorption, supporting its potential application in functional foods or dietary supplements (78). Besides, a systematic review identified consistent inverse associations between certain gut bacteria and glucose metabolism, including *Akkermansia muciniphila*, *Bifidobacterium longum*, Faecalibacterium, the *Clostridium leptum* group, and *Faecalibacterium prausnitzii* (79). Increased abundance of these microbial communities was associated with improved glucose metabolism and insulin sensitivity. In summary, these findings suggest that fluorotannins can improve glucose metabolism by regulating gut microbiota. This proposed mechanism involves the enrichment of beneficial bacterial taxa alongside the suppression of harmful species, thereby establishing a synergistic “phlorotannin-gut microbiota-glucose metabolism” axis.

### 4.2 Regulation of lipid metabolism

SCFAs mainly come from the microbial fermentation of dietary fiber, including acetic acid (about 60%), propionic acid (about 25%) and butyric acid (about 15%). They are the primary energy sources of colon cells and play crucial roles in intestinal health, immunomodulation and microbial ecology (80).

Dietary polyphenols, including catechins and anthocyanins, are known to enhance SCFA production. Similarly, phlorotannins from brown algae such as *Fucus vesiculosus* significantly elevate propionate and butyrate levels. Some extracts can also promote the growth of Bifidobacterium, of which can produce acetic acid and activate PPAR-α pathway, thus to enhance β-oxidation of fatty acids in the liver and reduce blood triglyceride levels (81, 82). Given the variable effects of different algal extracts, advancing purification techniques is an imperative for future study.

Beyond SCFAs, phlorotannin metabolites such as GA also ameliorate metabolic disorders. In mouse models of steatohepatitis (MASH), GA accelerated lipid metabolism via IRF6-mediated suppression of PPARγ, and directly activated AMPKα to alleviate NAFLD progression (83). Phlorotannins could also modulate gut microbiota structure by lowering the Firmicutes/Bacteroidetes ratio, which is often found elevated in obesity. Besides, Bacteroidetes further metabolize polyphenols into bioactive metabolites that improve cholesterol homeostasis (84, 85).

In summary, phlorotannins regulate lipid metabolism based on the gut microbiota by modulating microbial composition, promoting beneficial metabolites, like SCFAs and GA, as well as targeting key signaling nodes such as AMPK and PPARα. These mechanisms underscore their potential role in preventing and treating metabolic disorders such as obesity, NAFLD, and dyslipidemia.

## 5 Phlorotannins and their metabolic pathways: from intake to target interaction

Early human and *in vitro* studies have demonstrated that phlorotannins are extensively metabolized by colonic microbiota, resulting in the production of low-molecular-weight phenolic derivatives, which are detectable in urine and plasma (50). This highlights the crucial role of microbial transformation in their bioavailability and bioactivity. In line with this, *in vitro* gastrointestinal digestion and fermentation models have shown significant degradation of phlorotannin extracts during simulated digestion and colonic fermentation, further emphasizing the involvement of both digestive enzymes and gut microbial enzymes in phlorotannin metabolism (48). These transformations contribute not only to the release of bioactive metabolites but also to the modulation of gut microbiota composition. Specifically, brown seaweed extracts rich in phlorotannins have been found to promote the growth of beneficial bacteria such as Bifidobacterium and Lactobacillus (86), while simultaneously increasing short-chain fatty acid production during colonic fermentation. This suggests a metabolic cross-talk between phlorotannins and the gut microbiota. Recent reviews also confirm that oral phlorotannins undergo biochemical transformations mediated by digestive and gut microbial enzymes, including hydrolytic cleavage and reductive metabolism, which play a key role in their absorption and systemic effects.

To fully understand the metabolic effects of phlorotannins, it is crucial to investigate their entire journey from intake to target interaction. The process begins with phlorotannin ingestion, followed by microbial transformation in the gut, where gut microbiota break down complex polyphenols into bioavailable metabolites. These metabolites are then distributed throughout the body, reaching target organs like the liver, adipose tissue, and muscles, where they exert their regulatory effects on glucose and lipid metabolism. The final stage involves target interactions, where these metabolites influence key metabolic pathways, such as insulin signaling and lipid oxidation. The Table 3 below provides a clear overview of this process, helping to clarify the complex role of phlorotannins in metabolic regulation.

**Table 3:** A sequential process of intake, microbial transformation, metabolite distribution, and target interaction.

Stage	Process description
Intake	Phlorotannins travel through the esophagus and stomach
Small intestine	Partially absorbed in the small intestine, but due to their high molecular weight, they are not fully broken down into small phenolic acids
Colon transformation	The majority of phlorotannins proceed to the colon, where they undergo microbial transformation into smaller bioavailable metabolites

Microbial transformation		Metabolite	Target interaction	Metabolic regulation	References
Enzymes	Gut microbiota				
Polyphenol oxidases, polyphenol hydrolases	*Bifidobacteriu, Bacteroides*	Gallic acid	FXR signaling	Enhance intestinal barrier function	(102, 103)
Polyphenol oxidases	Entterococcus	Gallic acid	PTP1B	Enhances insulin sensitivity and fat oxidation	(104, 105)
Hydrolases, dehydroxylases	*Lactobacillus*	Gallic acid	AMPK	Inhibit hepatic gluconeogenesis	(48)
Deaminases	*Bacillus, Enterococcus*	2,3-dihydroxybenzoic acid	NF-κB	Potential effects on the intestinal barrier and inflammatory pathways	(106, 107)
Fermentation enzymes	*Bacteroides, Lactobacillus*	Short-chain fatty acids (SCFAs)	GPR47 GPR41	Modulates gut microbiota, improves metabolic health	(108, 109)
β-glucosidases, dehydroxylases	*Clostridium*	Phlorotannin metabolites (incl. Dieckol derivatives)	AMPK	Promotes energy homeostasis and lipid breakdown	(75, 108)
β-glucosidases	*Lactobacillus, Bifidobacterium*	Gallic acid	PPARα/PPARγ	Accelerate lipid metabolism and delay NAFLD progression	(110, 111)

## 6 Clinical evidence and translational potential of phlorotannins in metabolic disorders

### 6.1 Preclinical and clinical studies

Diabetes is a metabolic disorder characterized by elevated blood glucose levels. One effective treatment is to inhibit enzymes responsible for carbohydrate digestion, thereby reducing postprandial blood glucose level (87). In this context, extracts from brown algae such as *Undaria pinnatifida* have shown promise. *In vivo* studies demonstrate that *U. pinnatifida* extract lowers fasting blood glucose in diabetic mice by modulating key genes, including upregulating Pi3k, Glut4, Akt, and Ampk while downregulating Foxo1, Pgc-1α, Gsk-3β, and G6pc (42).

Different brown algal extracts appear to inhibit α-glucosidase through distinct mechanisms, depending on their composition and molecular weight profile (88). For instance, *Laminaria japonica* extract acts as an effective α-glucosidase inhibitor, potentially limiting intestinal monosaccharide release (89). Network pharmacology analyses further suggest that phlorotannins may target multiple proteins implicated in type 2 diabetes, such as BACE1, AKT1, ESR1 to regulate glucose metabolism (90). Clinically, a meta-analysis confirmed that brown algae supplementation significantly improves glycemic control, reducing postprandial glucose, HbA1c, and HOMA-IR, with higher doses (≥1,000 mg) conferring greater benefits (91). In addition, a double-blind randomized trial found no sex-specific differences in the glucose-lowering roles of phlorotannins (92).

To date, several randomized controlled trials (RCTs) have explored the dose–effect relationship of phlorotannins in the regulation of glycolipid metabolism in humans, providing preliminary evidence for effective dose ranges and safety margins. In a double-blind RCT involving individuals with prediabetes, a single oral dose of 600 mg *Ecklonia cava* extract (containing approximately 13% phlorotannic polyphenols) significantly attenuated postprandial glucose responses without reported adverse effects (93). According to safety evaluations summarized by the European Food Safety Authority (EFSA), daily intakes of *E. cava* phlorotannins up to 263 mg in adults (163 mg/day for adolescents aged 12–14 years; 230 mg/day for adolescents >14 years) are considered safe when used as dietary supplements (94). Within this regulatory range, a 12-week randomized controlled trial in 97 overweight adults demonstrated that supplementation with *E. cava* polyphenol extract at doses of 72 mg or 144 mg/day significantly reduced total cholesterol, LDL-C, and the total cholesterol/HDL-C ratio in a dose-dependent manner (95). Similarly, a 12-week randomized controlled trial in patients with hyperlipidemia showed that daily supplementation with 400 mg of a polyphenol-rich *Ecklonia cava* extract produced comparable lipid-lowering effects (96). From a safety perspective, *E. cava* phlorotannins have a long history of use as food supplements. In the United States, supplements containing *E. cava* phlorotannins have been marketed since 2006, typically providing approximately 100 mg/day (94). Although concerns have been raised regarding iodine content in brown algae extracts—particularly for individuals at high risk of thyroid dysfunction—no direct toxic effects attributable to phlorotannins themselves have been reported to date.

Taken together, current evidence suggests that the metabolic effects of phlorotannins are dose-dependent but context-specific (48), influenced by extract composition, dosing duration, baseline metabolic status, and population characteristics. While effective and safe dose ranges have been proposed for adults and adolescents, inconsistencies across studies underscore the need for larger, well-controlled clinical trials employing standardized phlorotannin preparations and multiple dose levels. Such studies will be essential to refine the optimal dose window, clarify inter-individual variability, and support the clinical translation of phlorotannins for glycolipid metabolic regulation.

### 6.2 Translational challenges in clinical application

Growing evidence support the beneficial role of phlorotannins in modulating glucose and lipid metabolism. Although PTs was discovered nearly 50 years ago (1978) (97), there are still key gaps in metabolomics mechanism, large-scale clinical verification and comprehensive safety, which hinders their large-scale production and clinical transformation.

Current research on phlorotannins suffers from limitations. Most studies merely document phenotypic improvements in metabolic parameters, like blood glucose and lipids level, but lack mechanistic depth. In addition, as the comparative data is limited, the potential benefits of combination regimens have not been studied. More critically, the fundamental differences in the liver enzyme system and renal excretory function various from species make it impossible to direct animal experimental data into humans. Additionally, animal models of metabolic diseases are typically artificially constructed with single etiologies (6), whereas human metabolic disorders naturally present with multiple comorbidities, such as hypertension, insulin resistance, and inflammatory responses, which involves far more complex pathological mechanisms.

Consequently, systematic clinical research has become an indispensable prerequisite for the clinical application of phlorotannins. Besides, formulation development represents another critical bottleneck. While most animal studies use crude extracts or highly purified monomers, human applications require pharmaceutical-grade formulations. Phlorotannins exhibit poor stability under environmental conditions such as temperature and pH variations, coupled with low water solubility and susceptibility to enzymatic degradation *in vivo*, resulting in suboptimal bioavailability (92). These challenges demand process optimization, including the selection of appropriate excipients, refinement of preparation methods to enhance stability, and the adoption of advanced delivery technologies such as ultrasound-assisted extraction (USAE) (98), microspheres, hydrogels, and nanoparticle-based systems (99) to improve solubility and *in vivo* delivery efficiency.

Bridging the gap between promise and clinical practice requires future research to overcome challenges in mechanism elucidation, clinical validation, and formulation science, ultimately confirming phlorotannins as a viable adjunct therapy.

## 7 Current research gaps and future perspectives

Marine organisms are a rich source of diverse phenolic compounds, among which phlorotannins have gained increasing research interest due to their unique chemical structures and potential to modulate gut microbiota and host metabolism. However, in order to accelerate its clinic application, some research gaps still need to be solved.

First of all, the metabolic role of phlorotannins compounds in different individuals are not yet clear; for example, health population, patients with prediabetes or diabetics. It is unclear whether their influence on gut microbiota or metabolic pathways differs according to host characteristics or disease stage. In addition, the long-term safety profile of phlorotannins have not been systematically established. Most studies use single doses or narrow doses range, which make cumulative effects of long-term intake on metabolic homeostasis unclear. A further limitation is the scarcity of publicly available intervention-based metabolomics datasets (in humans or animals) following phlorotannin supplementation. This gap constrains systematic mapping of *in vivo* biotransformation products, tissue distribution patterns, and downstream pathway engagement, thereby limiting evidence-weighted identification of novel targets for Figure-level mechanistic integration.

A major translational challenge lies in the low bioavailability of phlorotannins (100). These compounds are prone to degradation under environmental and gastrointestinal conditions, and their large molecular size limits intestinal absorption. Although the newly delivery system and improved extraction methods show the improved stability and accuracy, most of them are lack clinical verification.

From an application standpoint, phlorotannins are typically consumed indirectly through dietary brown algae, and there is no standardized or collaborative application at present. Besides, there is limited research on its combined effect with dietary fiber, probiotics or other bioactive ingredients, and individual variations in gut microbiota composition further complicate consistent efficacy.

In summary, future research should prioritize elucidating specific effects, establishing dose response and long-term safety data, advancing delivery technologies, clarifying functional distinctions from other polyphenols, and developing synergistic or personalized application frameworks to fully realize the potential of phlorotannins in metabolic health.

## 8 Conclusion

In summary, phlorotannins compounds represent a promising field in marine natural product research, and increasing evidence shows that they play an important role in regulating intestinal flora and human metabolism. Although their potential is considerable, this review also reveals significant knowledge gaps that must be addressed to unlock their translational value. Future efforts should leverage advanced analytical and multi-omics technologies to fully elucidate their mechanisms, biotechnological applications and ecological roles.
